# Assertive, Selective, Scalable IoT-Based Warning System

**DOI:** 10.3390/s22031015

**Published:** 2022-01-28

**Authors:** Ion-Dorinel Filip, Cristian-Mihai Iliescu, Florin Pop

**Affiliations:** 1 Computer Science and Engineering Department, Faculty of Automatic Control and Computers, University Politehnica of Bucharest, 313 Splaiul Independentei, 060042 Bucharest, Romania; dorinel.filip@upb.ro (I.-D.F.); cristian.iliescu97@stud.acs.upb.ro (C.-M.I.); 2 National Institute for Research & Development in Informatics, 8-10 Bulevardul Mareșal Alexandru Averescu, 011555-Bucharest, Romania

**Keywords:** warning system, IoT, MQTT, Raspberry Pi, scalability, selectivity

## Abstract

With the evolution of technology, developed systems have become more complex and faster. Thirty years ago, there were no protocols or databases dedicated to developing and implementing IoT projects. We currently have protocols such as MQTT, AMQP, CoAP, and databases such as InfluxDB. They are built to support a multitude of data from an IoT system and scale very well with the system. This paper presents the design and implementation of an IoT alert system that uses MQTT and InfluxDB to collect and store data. We design a scalable system to display assertive alerts on a Raspberry Pi. Each user can select a subset of alerts in our system using a web interface. We present a bibliographic study of SoTA, the proposed architecture, the challenges posed by such a system, a set of tests for the performance and feasibility of the solution, and a set of conclusions and ideas for further developments.

## 1. Introduction

The IoT (Internet of Things) is evolving like never before. Developers and scientists are working together on solutions that could make our lives easier and daily processes faster and more efficient [1]. With such technological development, projects that seemed difficult to achieve are becoming more and more feasible, being one of the most important factors in development in medical prevention [2].

Having a normal daily routine might sometimes be challenging for people with various physical conditions asking them to monitor different environmental parameters. A portable device that could alert them each time a life-threatening or unusual event occurs would be a great advantage [3].

In this article, we approach the design, implementation, and evaluation of an application designed to help people that need to check constantly specific environmental parameters to receive timely and reliable warnings. These parameters could be referring to local measurable values (such as temperature, humidity, geomagnetic energy, pressure) or data coming from specific sources (such as data about solar flares). A device that could communicate with such sensors or external real-time data sources of sensors would come to their aid.

We design our solution to be easy to setup (even for somebody not used to the technology) and part of an efficient, scalable cloud-based solution. Our main goal is to make people’s lives easier through technology. We know that every time it comes to a person’s health, things must be as accurate and fast as possible. In some cases, every second counts, which is why we design our application as a real-time cloud architecture.

Our use-case of delivering tailored warnings for users with special needs differs from any traditional warning system (such as a fire alarm) for generating warnings that might not be important for everybody in a room or a building but a specific individual only. We name that an assertive warning system and aim to make it bold in the eyes of the interested user and non-disruptive to other activities.

The main objectives and requirements for our early warning system can be summarized as follows:

- The system should be easy to setup even for a non-technical person;
- The system should be mobile and free to roam between multiple network, without a static address;
- The system should consider both locally collected data and data from external sources;
- Locally collected data should be stored efficiently for further analysis;
- The user should be able to customize which alerts to receive;
- The alerts should be delivered in real time;
- The system should be secure;
- Open standards should be used where applicable;
- The entire system should be efficient and scalable.

In this paper, we propose an architecture with implementation details for a selective alerting device. The device used is a Raspberry Pi, and the configuration is done remotely from a website. We consider data retrieved from an external weather website and data from local sensors connected to the Raspberry Pi as use-cases.

The sensors data are stored in a time-series database specialized in working with time-related data such as the evolution for temperature or atmospherical pressure. In order to have a graphical representation of the data, we use a web application called Grafana. This application takes the database as a data source and offers the functionality of customizing dashboards. These dashboards can be customized to represent different data by writing a query to the database.

Considering the requirements of embedded/IoT devices, working with energy and sometimes other resources (such as CPU, RAM, and storage) as a premium, using the suitable communication protocols for each scenario is crucial. In addition, storage and analysis of a large amount of data (which we expect from the sensors) ask for suitable database management solutions.

Based on our experience with multiple communication protocols (including CoAP, MQTT, AMQP, JMS, REST, and XMPP) and the fact that using a relational database to store a very large amount of data would result in both a slow down the query time and a considerable storage overhead, we decided to present a solution which uses MQTT as a lightweight communication protocol and a NoSQL database (called InfluxDB) as data storage.

Using MQTT as a communication protocol allows us to easily achieve lightweight asynchronous communication between the cloud and mobile devices. At the same time, InfluxDB fits the need of efficiently storing large amounts of time-series data while keeping a reduced query time and a high insert throughput (for collecting data for multiple devices).

Even though the entire use-case can be described in a few words, the problem includes multiple hidden challenges. In our article, we present the multiple challenges as well as a unified solution for most of the challenges, using well-known protocols and open-source solutions [4].

In our technical paper, we present the detailed solution as well as some experimental results proofing the fitness and the scalability of the solution.

Following the introduction, the paper is structured as follows. In Section 2, we present state of the art. We concentrate our focus on the functional components for our use case. The reader should refer to this section to better understand our choices and the properties of different used approaches. Section 3 presents the proposed architecture. Section 4 describes the prototype application in rich detail, including relevant code snippets. The performance results and their interpretation are presented in Section 5. The final section provides conclusions and future work.

## 2. State of the Art

In this section, we present the current level of development in the field and the possibilities that technology offers us at this time. We do an analysis on other people’s research about the technologies we use in our architecture.

### 2.1. Health Monitoring Device

OneCare [5] is a medical device built by a research team om Portugal. This device aims to monitor people with various health conditions, especially the elderly. This device notifies the family and the doctor who cares for the person if something unusual happens. The device monitors the pulse, connects via Bluetooth to various measuring devices such as a scale or a blood glucose meter, and tracks the person’s physical movement, being able to issue an alert in case the person falls accidentally or due to a crisis. Such a system of acquisition generates a constant flow of data. OneCare uses MQTT message communication protocol with Mosquitto (https://mosquitto.org, accessed on 27 November 2021) as a broker implementation. This protocol is very suitable for IoT acquisition systems and can manage many messages from many clients.

### 2.2. Early Warning System

There are early warning systems (EWS) that are designed to generate alerts in case of various extreme conditions like natural disasters or ecological threats. Poslad et al. [6] presents in his paper an architecture for such a system. They use AMQP (Advanced Message Queuing Protocol) as a message exchange protocol which, like MQTT, is an asynchronous messaging protocol [7]. Both protocols use publish/subscribe pattern of communication [8,9]. MQTT is proving to be faster in terms of latency in terms of performance in the Internet of Things systems. This increased speed is due to the small size of the message and the header but also to the reduced flexibility. AMQP has increased support for security, reliability, and provisioning, while MQTT aims to be a simpler message exchange protocol [7,10,11]. We find MQTT more suitable for our use case for our project, and we use JWT tokens for stateless authentication and session management. In addition, due to the low use of bandwidth and small message overhead, we find MQTT more suitable for embedded devices having power usage as a premium [12,13].

### 2.3. IoT Security

Paramveer Singh and Monika Sharma proposed a network architecture model used by an IoT application to help reduce the number of DDoS and MIRAI attacks [14]. They propose the use of two network channels in applications. For the main network channel, all the IP addresses of the connected devices are visible, while the second one has the role of masking the IP addresses in front of an attacker. The communication between the two channels is achieved through encrypted messages, thus preventing an attacker from easily finding the IP addresses.

In our proposed solution, we secure our fog network by changing all the active connections to the devices into client-server ones. With this approach, knowing the IP addresses of the mobile devices is exploitable by an attacker since no network ports will be listening for new connections.

MQTT is a lightweight and low latency messaging protocol [12]. It uses the publish/subscribe pattern and topics for publishing messages. This way, a client publishes a message to a topic and any other client subscribed to that topic will receive it [15].

**Figure 1:**
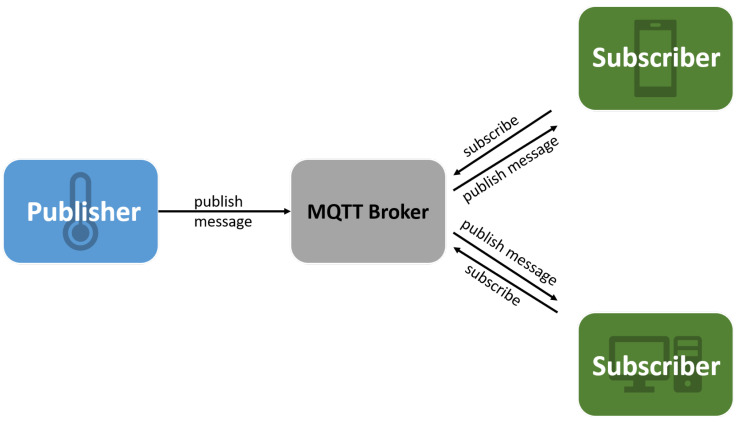
MQTT general components schema and workflows.

In Figure 1, we present the main components and workflows in MQTT.

We use MQTT as a communication protocol to have a real-time delivery of the data from all these sensors and trigger alerts as fast as possible. In this protocol, each message consists of a topic and a payload. We use the topics to differentiate various data flows.

An important feature of this protocol is quality of service (QoS), which allows the client to choose the best approach to network reliability.

It can be configured on three levels of QoS [16,17]:

- QoS 0—nothing is guaranteed;
- QoS 1—each message reaches target at least once;
- QoS 2—each message reaches target exactly once.

The selection of QoS is made based on the system’s needs. For example, if there is a need for predictive data delivery, QoS 2 is the way to go. QoS 0 is used when missing the messages has no significant impact on the system. If the system can easily tolerate duplicate messages, QoS 1 is used.

MQTT is designed for M2M (machine to machine) communication and runs over TCP. It has a small packet size which enhances the latency and overhead. Data sent over this protocol can be in any form, so it supports JSON, XML, text, or binary data [18]. Only three components are needed to create an application using MQTT: publisher client, broker, and subscriber client. There are several open-source MQTT brokers, Mosquitto [17] being one of the most popular ones.

### 2.4. JWT and MQTT

In order to increase the security of our application, we need a good method to authenticate the Raspberry Pi on the server. We need a method that is both fast and secure. Once authenticated or registered as a publisher, a device can send data until shutdown. The architecture developed by Adhitya Bhawiyuga et al. (2017) [19] presents a solution to the situation where an attacker uses the fact that once authenticated, a device is no longer verified for its authenticity and therefore, in the same session, a villain can send any data using a hijacked session. Preventing TCP session hijack using TLS/SSL is an option. However, Adhitya Bhawiyuga et al. propose a better approach using token-based authentication and MQTT to get much better session management and a good way of checking the sender’s authenticity. The technology they choose is JSON Web Token. A JWT token is a signed JSON object which contains a claim together with some other fields, enabling the receiver to cryptographically authenticate the sender/issuer of the request [20]. Using JWT tokens, we achieve better session management and improvement of security.

If we do not involve session management, a client should authenticate each request by sending the login credentials (represented by username and password) with every request. Moreover, the server should repetitively check them against the database.

In a JWT based solution, like the one we chose, a token is sent to the client after login if the credentials are correct. The received token has an expiration time and, while it is valid, can be used for posting data on the MQTT topic. There is also an improvement in performance because there is no need to check the username and password by querying the database if the token is valid. It can also contain other authenticated pieces of information about the client.

### 2.5. InfluxDB

Time-series databases are NoSQL databases designed to work with high loads time series of data retrieved from various systems. Thus the data these databases work with is timestamped data retrieved from measurements devices. This kind of data, like temperature, pressure, blood pressure, solar radiation, wind speed, and so on, has value for further analysis only if it has a corresponding timestamp. Any discrepancy between data and timestamp can cause a misunderstanding of information.

SQL databases are flexible and easy to work with, but continuous big loads of data represent the problem in IoT systems. Relational databases have full support for ACID (atomicity, consistency, isolation, durability) while NoSQL databases support BASE (Basic Availability, Soft-state, Eventual consistency) [21]. This difference is important in the matter of performance. How each database model manages, transactions had a direct impact on the performance. ACID is useful when you want to put the worst-case scenario first, and also data consistency is essential. On the other hand, BASE guarantees consistency in the future; it might be immediate or when the data are read. Scalability is a great challenge for relational databases due to their method of storing indexes and the big necessity of hardware to process the data [21]. Adding more hardware is not always the optimal solution; things might just work better in another way. InfluxDB is a NoSQL database optimized for working with time-series data. Several pieces of research have shown that it is suitable for IoT systems that produce high loads of data that need to be stored and analysed [22,23,24]. It supports SQL-like (InfluxQL) queries but offers optimized performance for time series.

### 2.6. Conclusions and Decisions

The architecture we propose combines several IoT-friendly technologies (including MQTT, JWT, and InfluxDB) to obtain a secure and fast alerting device. We aim to make life easier for everyone who needs this information daily.

Based on the biographical study, we decided on the protocols and technologies considered for the study. We made the following conceptual analysis of other existing IoT platforms and we made a comparison with our proposed solution (see Table 1).

The adopted protocols and technologies come to serve the functional aims of our system, while keeping the entire solution affordable and scalable. For the data collection part we chose MQTT for its ability to delever asyncronous comunication with a small overhead. REST API via HTTPS is our choice for client-initiated transactions (other than the ones pushing new IoT data), while InfluxDB offers us scalable and efficient storage for large datasets. We use a SQL database for keeping the status of the system. The exact technology behind the SQL database (e.g., MySQL or PostgreSQL) is interchangeable through the ORM (Object–relational mapping) we use. A more detailed analysis of how we involve each of the chosen patterns/technologies is presented in the following sections.

## 3. Proposed Architecture

**Figure 2:**
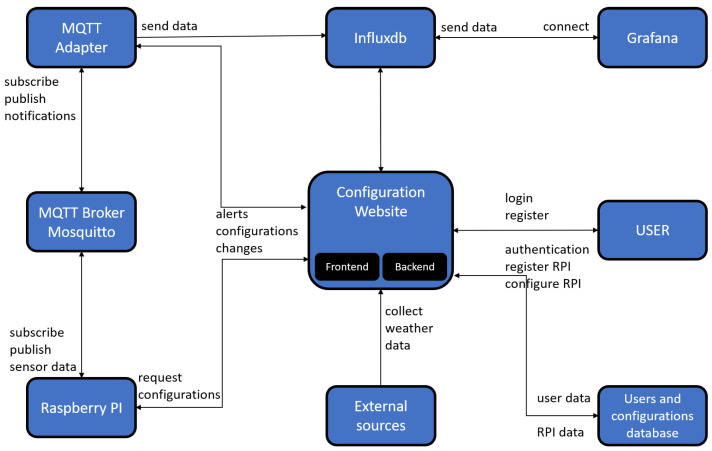
An overview of the architecture with the main blocks and interaction between them.

In Figure 2, we present the proposed high-level architecture for our solution.

We made this design in order to achieve performance and scalability. In Section 3.1 we present the architecture with implementation details.

### 3.1. Blocks Details

The *configuration website* module consists of both the backend and frontend, each of the components having an important role. Through the *frontend*, a user can register a new account, log in, add a new device to his/her profile or modify the settings of a device. The *backend* does all the work behind the user interface. It has multiple tasks to do. First, it has to gather weather and solar activity data from external sources and notify the corresponding devices if there is any life-threatening situation. Secondly, it keeps everything in order; Each active device is assigned to a specific user, so no other entity should have the right to access it or modify its settings. The backend keeps track of everything and ensures such situations are not possible.

The *Raspberry Pi* has two jobs. The first one is to alert the user if an alert is received. The second is to collect data from *BMP180* sensor and publish it to the *MQTT Broker*. Alerts are generated by the web backend and sent via MQTT to the RPI. This way, the RPI is not making continuous requests to the server and only when a special situation occurs. Alerts are of kinds: weather alert or configuration update alert. The RPI has an MQTT client who decides which type of alert has been received and requests the corresponding data from the server. Sending BMP180 sensor data to the *InfluxDB* database is done through an MQTT topic. The data are formatted in a JSON payload which is then formatted again by the MQTT Adapter. We present the data format in Section 4.

The role of the *MQTT adapter* is to filter the data and send it to the correct destination. We strongly consider that MQTT is the appropriate protocol for this task because of its many benefits. It is lightweight and uses a publish/subscribe model. On the other hand, HTTP uses the request/response model, which decreases performance and increases bandwidth usage. In addition, high consumption of resources drain the battery faster, and when you have to design and architecture for an IoT application, every resource is very important, including the battery usage [18].

In order to process all the data that IoT systems generate, we need auto-scaling and highly accessible time-series databases. NoSQL databases like InfluxDB are progressively used in this industry because it can manage big loads of data and also suits the requirements we mentioned before [22].

The *users and configurations database* keeps all the information about users and the configurations of each device.

The server generates a *JWT Token* and sends it to the entity that requested it each time it receives a valid username-password combination. It has a connection with the main database and queries it each time to verify the validity of the credentials. If the combination is correct, is generated a unique token that contains several pieces of information about the entity that requested it and sends it back to it. The token can then be used to make requests to the server and to publish data on an MQTT topic. No one can publish data or communicate with the server without a valid token. The validity of the token varies depending on the entity that requested it. A user who connects to the configuration website has a valid token for a shorter time than the token that a device uses to publish data on an MQTT topic.

### 3.2. Flows

In the following subsections, we present the most important flows in the functioning of the application.

#### 3.2.1. New User Account and Register an RPI

To register, the user has to fill a form that includes the following fields: Username; Email; Password; Confirm password. After a user gets our product, the first step is to make an account from which they are able to control the RPi; when a new account is created, the backend posts all the information about the user (name, email, password, profile picture) in the Users and Configurations database. After that, the user can log in and add the device to his/her account.

Each device can be activated only once to ensure that no device is added to more than one account only once. If someone has our device and can not activate it, then the person has to contact us in order to confirm the possession of the device and re-activate it.

After successfully adding the device to the account, the user can configure the following things: the type of alert that he/she wants to receive and the location of the RPI in latitude/longitude coordinates.

Initially, the RPI subscribes to ALERT/ topic, but once activated, it communicates with the MQTT Adapter only on its own topic. This way, the adapter always knows from whom each message is. The RPI does not need to make regular HTTP requests to the backend to check if there are new settings. Whenever an update is made to a certain RPI configuration, the backend sends an MQTT message to the RPI notifying a configuration update; when such a message is received, the RPI requests the new settings from the server.

#### 3.2.2. BMP180 Sensor Data to InfluxDB

**Figure 3:**
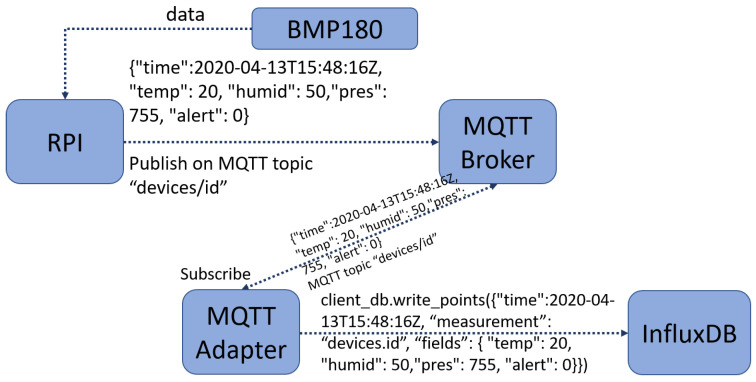
A representation of data flow between the BMP180 sensor and InfluxDB.

In Figure 3, we present a high-level schema of the communication between the BMP180, the Raspberry Pi, and InfluxDB. The Raspberry PI receives data constantly from the BMP180 sensor about temperature and pressure in the current environment.

The data are sent via MQTT, and to be able to publish data on a topic, the device needs a valid JWT token. If the device does not have a token, it is the first time it publishes data, and an initial authentication is needed to receive the first valid token. After receiving a valid token, the RPI can publish data on the topic designated for this kind of information transfer. If the token is expired, then a new token is requested with the current expired token. This way, we ensure that no other device can request a token in the name of our device. There is an MQTT adapter on the server that is subscribed to all devices’ topics. When data are received, first, we verify the token validity and then proceed to process the data. The data are then inserted in the InfluxDB database for the corresponding device based on the topic’s name. Data received by the adapter are in JSON format but has a different structure than the one accepted by InfluxDB. The modification of format takes place also in the adapter. The JSON data are converted to the new JSON format, and also a small verification of data validity is done. For example, you cannot insert string data for temperature.

#### 3.2.3. JWT Token Request

**Figure 4:**
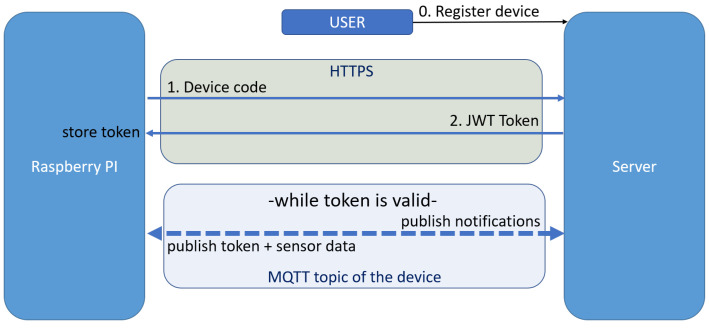
A representation of message handshake between the RPI and the server in the scope of authenticating the RPI.

As we previously described, each device needs to obtain a JWT token in order to be authenticable in the system. In Figure 4 we depict the handshake for the authentication.

The JWT token is the server assurance that the device sending data on a certain topic is actually it and not an attacker and helps the IoT device prove its session. We send the messages over HTTPS and MQTT between the RPI and the server. We use HTTPS for the first handshake of messages due to the protocol’s increased security and synchronous request-response flow.

To receive a token and be able to communicate with the server, a device first has to publish its unique code on the topic. The device can only do this operation once it is activated. Otherwise, the server will ignore its request. When the server receives a token request from an activated device, it generates a unique token and sends it back to the device. Once the device has a valid token, it can publish data from the sensors on the MQTT topic to be inserted into the database. The token has a limited availability time, and when it expires, The device can request a new one only if it has the old token. This way, we ensure that no one other than the device having the code in the request can ask for a token for that device. The server and the device are the only entities that know the expired token.

#### 3.2.4. Alert Notification

**Figure 5:**
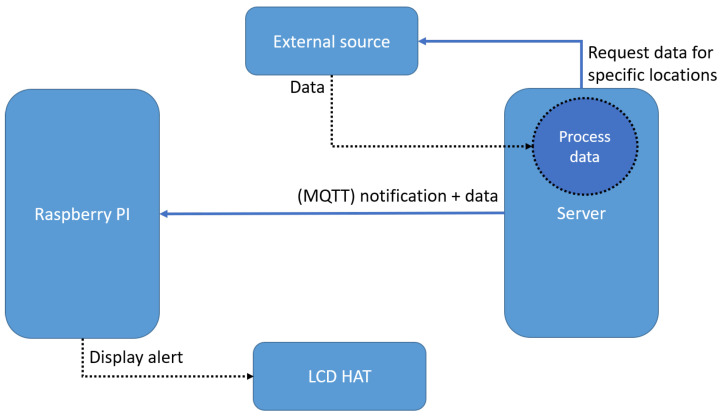
The message flow for alerts.

Sending alerts to the devices is an important part of the solution. In Figure 5, we present the data flow between the server and an external source and how alerts are sent to the Raspberry Pi.

The server requests data periodically from the external source to monitor the state of the weather. Then these data are processed, and for every device, the set boundaries of weather parameters are checked. If the system receives a value that is not in the chosen range, an alert is sent to the device. The alert is sent via MQTT, using a message containing the alert type and parameter value.

A single use-case is considered with the current prototype, and it comes with some predefined parameters (such as *min_temp*, *max_temp*, *min_press*, *max_press*. However, the database schema allows any parameter to be added to a device. Anybody interested in refactoring our idea can make business logic changes on the RPi with a few lines of code. A more general approach, offering higher flexibility, can be achieved using a tautology-based configuration.

#### 3.2.5. Raspberry Pi Settings Update

**Figure 6:**
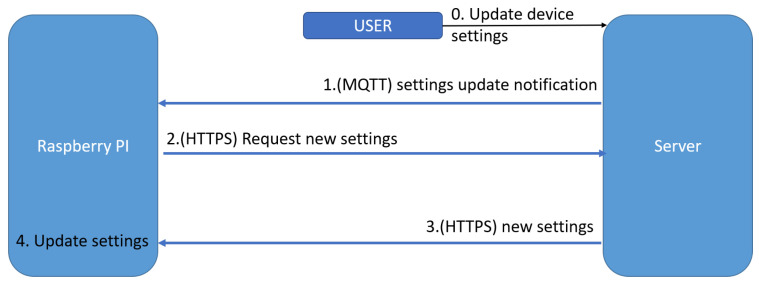
This schema represents the process of updating the settings for a device.

In Figure 6, is presented the flow of updating the settings of a device. The process is initiated by the user who updates the configurations on the website. We want to have a real-time update of the device so the speed is the key in this flow as in all the others.

The process is as follows:

- The user updates the configurations of one of their devices;
- The server sends a notification to the device via MQTT. We use MQTT for this notification because it is fast, and we need only to inform the device that there are new settings available;
- The device receives the notification and makes an HTTPS request to the server asking for the new settings. We use HTTPS for this request and send back the settings because it is easy to use via REST API, and more importantly, is transactional.
- Finally, the device receives the new configurations and makes the necessary modifications;

Both alerts and configuration update triggers are delivered to the RPi through MQTT. In our implementation, the device differentiates between those two based on the payload of the received message. Using two different MQTT topics should also be an applicable solution.

## 4. Implementation Details

### 4.1. User Registration

The registration page contains a form for entering new user credentials. The form has the following fields: Username; Email; Password; Confirm password. Each field is checked for validity; we present the form validation in Listing 1. Once the form is completed with valid data, all fields are saved in the database. An important part is how we store the passwords of the users. Each password is encrypted using the *bcrypt* library. A snippet of how the password is encrypted is presented in Listing 2.

Listing 1 **Code for checking the username and email of a new user. No existing username or email can be used.**

class RegistrationForm(FlaskForm):

    ...

    def validate_username(self, username):

        user = User.query.filter_by(username=username.data).first()

        if user:

           raise ValidationError(’That username is taken.’)

 

    def validate_email(self, email):

        user = User.query.filter_by(email=email.data).first()

        if user:

           raise ValidationError(’That email is taken.’)

Listing 2 **Code for encrypting the password.**

hashed_password = bcrypt.generate_password_hash(form.password.data).decode(’utf-8’)

### 4.2. User Login

In order to control their devices, the user should log in to their account. To achieve that, the user should fill the credentials into a form. The credentials are securely sent through HTTPS, and the form data are then compared with the hashed data from the database. A snippet of the login route is presented in Listing 3.

To manage authentication status, we use the *flask login* library, which handles everything about user management. An essential function of this library is cookie-based session management. It has easy-to-use functions like *is authenticated* which make writing code for user session management easier.

Listing 3 **Code for checking the username and email of a new user. No existing username or email can be used.**

if current_user.is_authenticated:

          return redirect(url_for(’home’))

      form = LoginForm()

      if form.validate_on_submit():

        user = User.query.filter_by(email=form.email.data).first()

        if user and bcrypt.check_password_hash(user.password, form.password.data):

          login_user(user, remember=form.remember.data)

          next_page = request.args.get(’next’)

          return redirect(next_page) if next_page else redirect(url_for(’home’))

        else:

          flash(’Login Unsuccessful. Please check email and password’, ’danger’)

### 4.3. RPI Activation

A user must first add it to their account to activate an RPI. As presented in Section 4.7, each device has an *auth_status* flag which can be 0 for not registered and 1 for registered. Once activated and started, the Raspberry Pi sends its unique code to the server in order to receive an authentication JWT token. This flow is presented in Figure 4. The foreign key in the devices table has the role of identifying all user devices.

If someone brute forces a device’s unique code, the only way to re-activate it is to contact the application’s support team to confirm your identity and the fact that you are in possession of the device. If the unique codes are correctly generated, this situation is not likely to happen.

### 4.4. RPI Configuration

**Figure 7:**
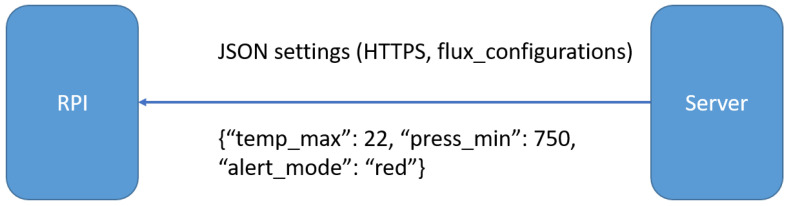
RPI configuration JSON message.

In Figure 7 is represented a regular JSON file received by the Raspberry Pi from the server. It contains several measurements names and values. The RPI expects a series of measuremens names and for each received one there is a corresponding local variable. The variables are updated to the new values.

### 4.5. Server Configuration

We first developed the project on a local machine, and then we built every component in a container.

The components that are running in separate containers are:

- MQTT Broker Mosquitto;
- MQTT Adapter;
- Grafana web interface;
- Configuration website;
- InfluxDB instance.

All these containers are deployed using a YAML file that keeps configurations for all services. Each container represents a service and has specific attributes like the networks through which it communicates with other containers and the open ports. In Listing 4 is presented as a part of the Docker stack file.

There are the configurations for the *MQTT Broker*, the *MQTT Adapter*, the volumes for *Grafana* and *InfluxDB* and the defined networks. The two MQTT components communicate through the *broker* network. The MQTT Adapter communicates with Grafana via *influxdb* network in order to insert the received data in the database.

Listing 4 **Part of the docker YAML Docker stack file.**

services:

  broker:

    image: eclipse-mosquitto

    ports:

      - "1883:1883"

    networks:

      - broker

 

  adaptor:

     image: mihaiiliescu4/mqttadapter

     environment:

      - INFLUXDB_HOST=influxdb

      - MQTT_HOST=broker

     networks:

      - broker

      - influxdb

 

volumes:

  influxdb:

  grafana:

 

networks:

  influxdb:

  broker:

### 4.6. Sensor Data Representation

Data from monitoring devices without a visual representation is hard to understand and, most importantly, to interpret. We collect data from the BMP180 sensor every 15 min from all connected devices. The data are formatted in a JSON string and sent to the MQTT broker on the specific topic to each device. To send the data to the MQTT broker, the RPi acts as an MQTT client who receives the data from the devices and publishes it on the topic.

Next, the MQTT adapter is subscribed to all devices and waits for data from every device. The adapter receives every JSON message from the devices, checks the topic the data was sent on, and then parses the data to be inserted in the InfluxDB database.

**Figure 8:**
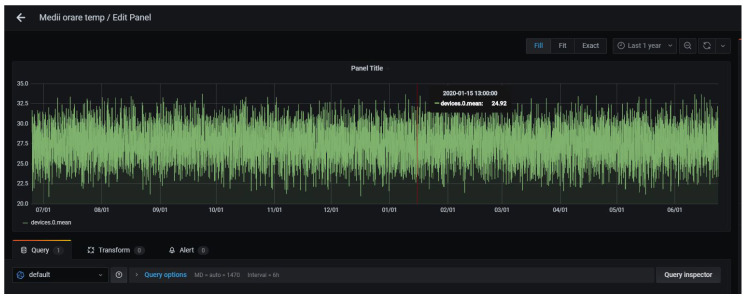
Representation in Grafana of the average temperature/hour in a year for a device.

Grafana [33] is a web platform designed for analyzing data. We include it into our stack to allow intuitive visualization of data. InfluxDB is one of the officially supported data sources for Grafana, and it is really easy to integrate. Using Grafana, we can design custom dashboards for monitoring the data from devices. Such a dashboard can be seen in Figure 8.

The SQL database deserves the application for the accounting and management of users and devices.

### 4.7. SQL Database Schema

**Figure 9:**
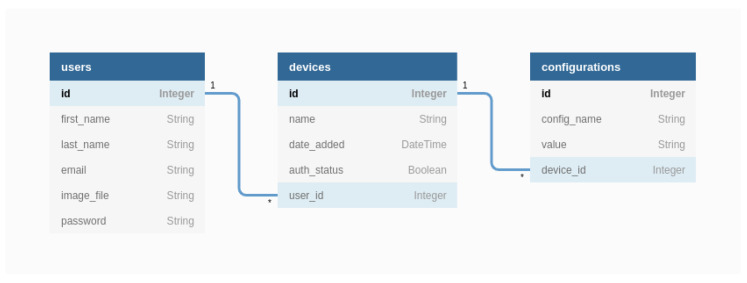
UML diagram of the SQL database.

In Figure 9, we present the UML schema of the SQL database used by the configuration server to manage both authentication and device configurations.

There is a one-to-many relationship between the users and devices tables and a one-to-many relationship between devices and configurations tables. Moreover, the configuration table does support adding generic configurations (identified by config_name for each of the devices). The database has this design for two reasons:

– First, in order to be able to add multiple devices to a user account, we need a one-to-many foreign key between the devices and users tables. The user id represents the foreign key, which is also present in the devices table.
– Second, creating a configuration table with predefined columns would make it difficult to add new configurations. For example, if the table has the columns temperature, humidity, pressure, and device id, and we want to add the column solar radiation, we have to change the table schema and add a column for the new entry. This means changing code, and also we have to support the cost of completing the column data for all the existing rows if needed. A better approach is to use only two generic columns for all configurations. A column for the configuration name and one for the value is enough for storing many configurations. The device_id column is for identifying the related device.

### 4.8. InfluxDB Database Schema

The way we represent the data inside InfluxDB is important since splitting uncorrelated data between measurements will optimize the query times, while keeping multiple - simultaneous measured values - into the same measurement will help optimize the space taken by InfluxDB. We chose to keep the data for each device in a measurement given by its ID (in devices.ID format). In Listing 5, we present the InfluxDB request for adding four values (temp, humid, pres, and alert) for device 99.

Listing 5 **Example of JSON inserted in InfluxDB.**

{

 ’time’: ’2020-06-24T00:43:10.409438Z’,

 ’measurement’: ’devices.99’,

 ’fields’: {

  ’temp’: 22.8,

  ’humid’: 78,

  ’pres’: 769,

  ’alert’: 0

 }

}

### 4.9. MQTT Adapter

The MQTT Adapter is a python script running in a container. It has the job to handle all published messages by the devices and insert the data sent by them in the InfluxDB database.

The Adapter first checks if the database exists. Otherwise, it creates is and then inserts the data in it. The messages received by the Adapter are in the simple key-value JSON format generated by RPi devices.

In Listing 6, we present a minimal Python 3 function that processes a message. The function formats the data and also inserts it into the database.

Listing 6 **The on_message function for the adapter.**

def on_message(client, userdata, msg):

    print(

        strftime("%Y-%m-%d %H:%M:%S", gmtime()) +

        " Received a message by topic [%s]" % msg.topic)

 

    # Create DB sensors if it doesn’t exists

    create_db_influx(client_db)

 

    m_decode = str(msg.payload.decode("utf-8", "ignore"))

    msg_json = json.loads(m_decode)

    fields = {}

    for k, v in msg_json.items():

        if is_number(v):

            fields[k] = float(v)

 

    date = msg_json.get("time")

    json_body = [{

        "measurement": msg.topic.replace(’/’, ’.’),

        "time": date,

        "fields": fields

        "fields": fields

    }]

    if date:

        json_body[0]["time"] = date

    else:

        json_body[0]["time"] = datetime.datetime.strftime(datetime.now(), ’%Y-%m-%dT%H:%M:%S.%fZ ↪ ’)

    client_db.write_points(json_body)

The format of the input JSON is the one presented in Listing 5.

## 5. Results

This section presents a set of experimental results aiming for the performance and feasibility proof for the proposed solution.

The section is organized as follows:

- In Section 5.1, we present the experimental setup used for running our experiments as well as the assumptions used for generating all the simulated data;
- In Section 5.2, we check the costs (time and storage for InfluxDB) considering a setup with up to 100 devices. This study is important to see both the scalability of the solution and the cost estimate;
- In Section 5.3, we set scalability limits for using the free version of the Open Weather API to serve alerts to interested users on weather parameters.

### 5.1. Experimental Setup/Environment

The tests were executed on a development server hosted by DigitalOcean (https://www.digitalocean.com, accessed on 27 November 2021). The details for the allocated virtual machine are those in Table 2.

For our experiments we generated random data assuming that each simulated device generates four values considering that:

- temperature is a float with one decimal place between 20 and 35;
- pressure is an integer between 740 and 770;
- humidity is an integer between 40 and 80;
- alert is a boolean (1/0).

### 5.2. InfluxDB Bulk Historical Data Load—Time and Memory Check

To assess the scalability and fitness of the chosen database (InfluxDB), we simulated a bulk migration of data from another database to InfluxDB, considering one year of data.

Since InfluxDB API allows multiple data points to be inserted through each REST API request, we decided to use a batch-size of 3500 points (sets of data containing four values) per request in order to avoid a considerable unrealistic overhead of making an HTTP call for each data-point.

**Figure 10:**
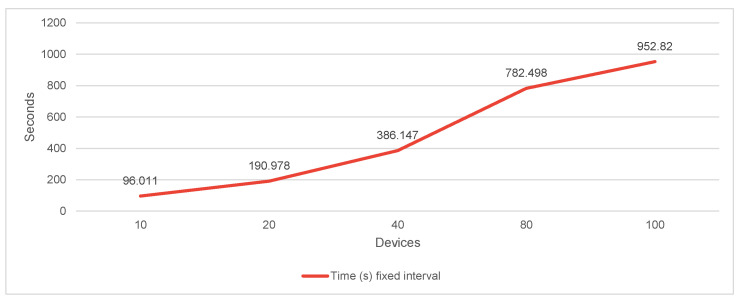
Elapsed time loading data for every 15 min for 1 year. Batch size 3500.

In Figure 10, we present the completion time for making the simulated migration while considering varying the number of devices.

Given the internal storage engine of InfluxDB, we can expect that it better optimizes timestamp storage and offers better performance when all the timestamps are equally spaced. For this reason, we experimented with two different scenarios:

- Fixed time interval—considering that the distance (in time) between all the points is always the same;
- Realistic time interval—adding a small delay or anticipation each time we generate a new timestamp in our simulation.

**Figure 11:**
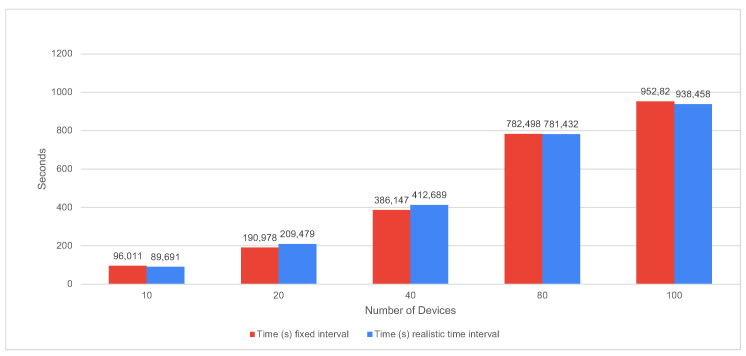
Elapsed time loading data for every 15 min for 1 year using equally spaced vs realistic spaced timestamps.

In Figure 11 we represented the bulk load of data for one year, considering both equally spaced and realistic spaced timestamps.

We can observe that—even though our expectation of having faster run time for equally spaced times is confirmed for most cases—we obtain feasible results for both versions, with a maximum of 952.82 s for data migration for 100 devices.

**Figure 12:**
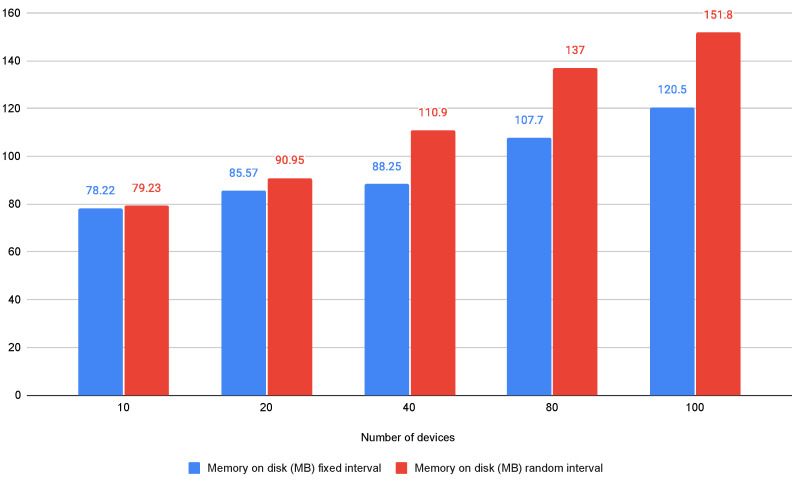
Storage space used by data generated for fixed time intervals vs data generated for random time intervals.

Another important cost factor for a database is the used storage space. In Figure 12 we represent the storage usage for both the cases.

We can observe a considerable difference between the memory occupied by data generated at fixed time intervals and that occupied by data generated at random time intervals, but even when having data for 100 devices for 1 year occupying around 150 MB which can be translated into a very cost-effective solution.

In Table 3 and  Table 4, we present a throughput analysis derived from our experiments. We notice that even using commodity resources, and the database can handle more than 3000 points per second with small performance variations.

Analysing the results, we can conclude that:

- The chosen database solution is fit for our use-case and offers great performance for a very small cost;
- Using an approximate timestamp (e.g., rounding all the timestamps to the closest minute) can result in better performance and a smaller storage footprint.

### 5.3. OpenWeatherMap API as an External Source of Data

Having an external data source usually comes with the downside of having limited throughput from it. In this section, we assess the feasibility of using the free plan of OpenWeatherMap as a data source.

OpenWeatherMap offers a free plan with a limited number of requests per minute. Their free plan offers 60 calls/min and 1,000,000 calls/month. With this free plan, if we make 60 calls per minute, we would exceed the limit. In Table 5 is represented the number of potential calls.

Considering that each device has a different location, We should make one request for each of them to refresh the status. In Table 6, we present the number of devices we can accommodate on the free plan if we use refresh times from 1 min to 25 min.

The conclusion comes easily: considering that the weather can not dramatically change in 5 min, we can accommodate up to 23 devices on the free plan, while on higher load, we can accommodate up to 694 devices by increasing the refresh time to 25 min.

Another important aspect while working with external data sources is the response time. To check how fast/slow OpenWeather will be for us, we simulated a scenario with 60 devices in 60 different locations. Since network conditions can really count, in this case, we repeated all the requests 20 times for each of the 60 locations. Since production API usually count on caches to serve the clients faster, we also considered two scenarios:

- Identical locations—keeping the same location for each device between iterations;
- Different locations—considering moving devices, randomly with random location changes between interations.

**Figure 13:**
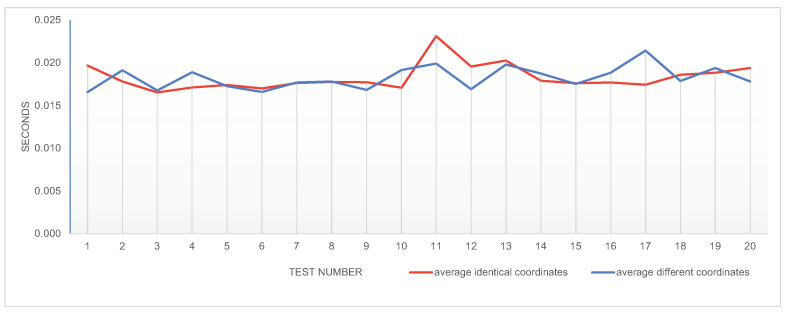
Side to side comparison of the average request response time per iteration of each case.

In Figure 13, we present the average time for each iteration for both of the scenarios.

Averaging the results, we obtain the average for the same coordinates as 18.299 ms and the average for different coordinates as 18.325 ms. There is no significant difference between the two scenarios, and the total times are really small. From the results, we can conclude that using such an external data source is feasible for our use case.

## 6. Conclusions and Future Work

The paper presents the design, implementation, and evaluation of an architecture serving an IoT device for selective alerting.

As main contributions, we presented the most important challenges and solutions that are relevant for the development of an IoT early-alerting selective device based on a long-running remote service; we presented the main existing solutions in the bibliography and made an evaluation of the main protocols for data queues; we proposed an architecture that combines communication HTTPS communication with MQTT to obtain optimal performance for each stream; we implemented a prototype for the proposed solution and presented the main implementation details; we tested the feasibility and scalability of the proposed solutions, focusing on the feasibility of data storage, the performance level of an external service (Open Weather API).

As future work in this field we aim for implementation of multiple data sources (to serve multiple use cases); firmware upgrade for RPi to allow more refined configuration of alert mode and how alerts are represented; adding objective comparisons between MQTT and AMQP, based on the current use case; characterization of the server-RPi communication delay.

The obtained experimental results allow us to say that our solution is feasible for both small and large deployments. We consider that the paper contributes to the common knowledge on designing an IoT architecture for selective alerting by providing an experimental-validated model of a minimal prototype.

## Figures and Tables

**Table 1:** Comparison of existing IoT data collecting solutions.

Platform	Collection	Analysis	Visualisation	Integration	Architecture	Storage	SLA Support
Kaa [25]	MQTT, CoAP, XMPP, TCP, HTTP, WiF, Ethernet, Zigbee	Real time	External Dashboard	Portable SDK, REST API	Server, Extensions, endpoint SDKs	MongoDB, Cassandra, HDFS, Oracle	Managed by user
ThingsBoard [26]	HTTP, MQTT, CoAP	Real time analytics (Spark, Kafka),	Web-based Dashboard	REST API, MQTT APIs	Layered RPC-based	PostgreSQL, Cassandra, HSOLDB	Managed by user
DeviceHive [27]	REST API, WebSockets, MQTT	Real time analytics (Spark)	External Dashboard	REST API, MQTT APIs	Microservice	PostgreSQL, SAP Hana DB	Managed by user
AWS IoT [28]	HTTP, MQTT, WebSocket	S3, Kinesis, Lambda, DynamoDB, Elastic Search	Web-based Dashboard	Connectivity, Authentication, Rules Engine, Dev Mode, Edge SDK	Cloud-Edge, Pipeline	Amazon S3	Prioritv Basis
Google IoT [29]	HTTP, MQTT	Dataflow, BigTable, BigQuery, Coding Service Engine	Web-based Dashboard	Connectivity, Authentication, Device Management	Cloud-Edge, Publish/Subscribe	Firebase	Prioritv Basis
Azure IoT [30]	HTTP, MQTT, AMQP, WebSocket	Stream Analytics, Azure Blob, Notification, Power Bl	Web-based Dashboard	Connectivity, Authentication, Device Management and Monitoring, IoT Edge SDK	Cloud-Edge, Client-Server with Azure Cloud	Azure SQL Edge, Azure CosmosDB, SQL DW, Storage Blob	Prioritv Basis
IBM Watson IoT [31]	HTTP, MQTT	Require Configuration	External solutions	Plugins in C#, C, NodeJS	Client-Server with IBM Cloud	DB2	Unknown
NetIoT [32]	HTTP, MQTT	Real-time	Dashboard	Device Gateway, RabbitMQ	Microservice	Cassandra, PostgreSQL	Managed by user
Our solution	MQTT	Real-time	Grafana	REST API, MQTT	Client-Server	InfluxDB, SQL	Managed by user

**Table 2:** Virtual machine configuration.

OS	Ubuntu 18.04 LTS (64 bit)
CPU	Intel(R) Xeon(R) CPU E5-2650L v3 @ 1.80 GHz
No. Cores	2 vCPUs
RAM	4 GB
Storage	50 GB Enterprise SSD

**Table 3:** Calculation of throughput. Batch size 3500.

Experiment	No Devices	No Points	Time (s)	Throughput (Points/s)
Fixed time interval	10	350,400	96.011	3649.581819
	20	700,800	190.978	3669.532616
	40	1,401,600	386.147	3629.705786
	80	2,803,200	782.498	3582.373373
	100	3,504,000	952.82	3677.50467
Realistic time interval	10	350,400	89.691	3906.746496
	20	700,800	209.479	3345.442741
	40	1,401,600	412.689	3396.262076
	80	2,803,200	781.432	3587.260312
	100	3,504,000	938.458	3733.78457

**Table 4:** Throughput average and limits.

Experiment	Average	Min	Max
Fixed time interval	3641.739653	3582.3734	3677.50467
Realistic time interval	3593.899239	3345.4427	3906.746496

**Table 5:** Number of total calls/month with 60 calls/minute.

Per Minute	Per Hour	Per Day	Per Month
60	3600	86,400	7,464,960,000

**Table 6:** Table of potential requests at different time intervals in order to not exceed the free limit.

Time Interval	1 min	5 min	10 min	15 min	20 min	25 min
Number of devices	23	115	231	347	462	694

## Data Availability

Not applicable.

## Abbreviations

AMQP	Advanced Message Queuing Protocol
CoAP	Constrained Application Protocol
DDoS	Distributed Denial-of-Service
EWS	Early Warning Systems
HTTPS	Hypertext Transfer Protocol Secure
IoT	Internet of Things
JMS	Java Message Service
JWT	JSON Web Token
M2M	Machine to Machine
MIRAI	a malware
MQTT	MQ Telemetry Transport
QoS	Quality of Service
REST	Representational state transfer
RPI	Raspberry Pi—a small single-board computers
TCP	Transmission Control Protocol
UML	Unified Modeling Language
XMPP	Extensible Messaging and Presence Protocol
